# 8-Hydroxy-2’-deoxyguanosine protein immunoexpression is associated with the pathogenesis of actinic cheilitis - Replay^⋆^

**DOI:** 10.1016/j.abd.2024.04.003

**Published:** 2024-07-08

**Authors:** Cíntia Barreto de Oliveira Varela, Cristianne Kalinne Santos Medeiros, Jabes Gennedyr da Cruz Lima, Éricka Janine Dantas da Silveira, Patrícia Teixeira de Oliveira

**Affiliations:** Postgraduate Program in Dental Sciences, Department of Dentistry, Universidade Federal do Rio Grande do Norte, Natal, RN, Brazil

*Dear Editor,*

The authors appreciate the comments on their article: “8-Hydroxy-2’-deoxyguanosine protein immunoexpression is associated with the pathogenesis of actinic cheilitis” and would like to clarify some comments regarding the article.^1^

When this study was carried out, the objective was to verify whether there was an association between 8-Hydroxy-2’-deoxyguanosine protein immunoexpression (8-OHdG) and the degree of morphological severity of actinic cheilitis (AC), since chronic exposure to ultraviolet radiation (UVR) is associated with the development and progression of AC to lip carcinoma. This fact led to the hypothesis that samples with higher degrees of dysplasia could harbor a higher expression of this protein. For this reason, cases of photoexposed healthy lips and photoprotected mucosa were not included in the sample. The inclusion of these two groups is suggested for further studies with comparison purposes with AC cases.

In relation to the sample patients who were smokers, it was not possible to observe a higher expression of 8-OHdG in the analyzed cases and this result may be due to the low percentage of smokers in the sample (7%).

According to the data from the present study, one cannot say that 8-OHdG can be used as a biological marker for the progression of AC to lip carcinoma. However, chronic exposure to UVR leads to the generation of reactive oxygen species (ROS) and, consequently, to the formation of 8-OHdG, which can cause mutations in cell DNA.^2^

In this context, 8-OHdG has been used as a marker of carcinogenesis in several experiments, being well-established as a prognostic factor in esophageal cancer.^3^ It has also been observed that this protein is overexpressed in actinic keratosis and Bowen's disease lesions when compared to adjacent unaffected tissues, healthy controls and squamous cell carcinoma (SCC) samples.^4^

The expression of the 8-OHdG protein can be observed both in the nucleus and in the cytoplasm since the main source of ROS generation is the mitochondria. This process leads to the oxidation of biomolecules, with consequent homeostatic cell imbalance, resulting in the development of several diseases such as atherosclerosis, diabetes, neurodegenerative disorders and cancer.^3^, ^4^, ^5^ Although the present study did not perform a differential analysis between nuclear and cytoplasmic levels of 8-OHdG, one can say, based on the literature, that increased levels of this protein may be related to the pathogenesis of AC.

## Financial support

None declared.

## Authors’ contributions

Cíntia Barreto de Oliveira Varela: Data collection, analysis and interpretation; Critical literature review; Preparation and writing of the manuscript, approved the final version of the manuscript.

Cristianne Kalinne Santos Medeiros: Data collection, analysis and interpretation; Statistical analysis, approved the final version of the manuscript.

Jabes Gennedyr da Cruz Lima: Data collection, analysis and interpretation, approved the final version of the manuscript.

Éricka Janine Dantas da Silveira: Study conception and planning; Effective participation in research orientation, approved the final version of the manuscript.

Patrícia Teixeira de Oliveira: Study conception and planning; Effective participation in research orientation, approved the final version of the manuscript.

## Conflicts of interest

None declared.
